# Academic Cross-Pollination: The Role of Disciplinary Affiliation in Research Collaboration

**DOI:** 10.1371/journal.pone.0145916

**Published:** 2016-01-13

**Authors:** Amar Dhand, Douglas A. Luke, Bobbi J. Carothers, Bradley A. Evanoff

**Affiliations:** 1 Department of Neurology, Washington University School of Medicine, St. Louis, Missouri, United States of America; 2 George Warren Brown School of Social Work, Center for Public Health Systems Science, Washington University in St. Louis, St. Louis, Missouri, United States of America; 3 Department of Internal Medicine, Division of General Medical Sciences, Washington University School of Medicine, St. Louis, Missouri, United States of America; Katholieke Universiteit Leuven, BELGIUM

## Abstract

Academic collaboration is critical to knowledge production, especially as teams dominate scientific endeavors. Typical predictors of collaboration include individual characteristics such as academic rank or institution, and network characteristics such as a central position in a publication network. The role of disciplinary affiliation in the initiation of an academic collaboration between two investigators deserves more attention. Here, we examine the influence of disciplinary patterns on collaboration formation with control of known predictors using an inferential network model. The study group included all researchers in the Institute of Clinical and Translational Sciences (ICTS) at Washington University in St. Louis. Longitudinal data were collected on co-authorships in grants and publications before and after ICTS establishment. Exponential-family random graph models were used to build the network models. The results show that disciplinary affiliation independently predicted collaboration in grant and publication networks, particularly in the later years. Overall collaboration increased in the post-ICTS networks, with cross-discipline ties occurring more often than within-discipline ties in grants, but not publications. This research may inform better evaluation models of university-based collaboration, and offer a roadmap to improve cross-disciplinary collaboration with discipline-informed network interventions.

## Introduction

For at least half a century, scientific activity has been characterized by the growth of team science. The size of scientific teams has increased across all scientific fields and disciplines, and teams have greater impact than individual scientists. “…sole authors did produce the papers of singular distinction…in the 1950s, but the mantle of extraordinarily cited work has passed to teams by 2000” [1] (p. 1038). Partly due to this seismic shift in how scientific activities are organized, a new discipline has emerged called the Science of Team Science (SciTS) [2]. A research agenda has been proposed for SciTS that includes important questions and challenges organized at multiple levels, including micro (role of individual scientists), meso (team characteristics), and macro (organizational and societal influences on science) [3].

One major research question domain in SciTS is the importance and influence of disciplinary dynamics and team science [4]. Although there appears to be a consensus around the idea that the most important scientific and public health challenges require new types of interdisciplinary collaborations and transdisciplinary concepts and methods [5,6], we are just at the beginning stages of understanding how to measure transdisciplinarity, let alone judge its value [7]. Bibliometric and survey approaches have studied interdisciplinary collaboration through analyzing publication patterns and scientists’ perceptions [8, 9, 10]. These studies suggested that certain disciplines excel in cross-discipline work (e.g., biology)[8], while others retained disciplinary boundaries while touting a multidisciplinary reputation (e.g., nanoscience)[11]. However, the role of discipline in relation to other contextual influences on transdisciplinary collaboration and productivity [12,13], particularly wider network factors, needs further study.

The general goal of this paper is to explore how disciplinary patterns among a community network of scientists are related to the likelihood of scientific collaboration. Specifically, we examine whether the discipline-discipline pairing, regardless of if they are the same or different (e.g., clinical-clinical or clinical-basic science), is independently related to whether two scientists collaborate. For example, suppose one researcher is a MD professor in a clinical specialty and a second is PhD assistant professor in social sciences. Does the clinical-social science pairing predict their tendency to collaborate on a grant or publication when other characteristics (degree, rank, network position) are taken into consideration? This question and analysis is a wider look at the relationship of discipline and collaboration that informs the agenda to support transdiciplinary science and infrastructure development.

The context for this study is the Institute of Clinical and Translational Sciences (ICTS) at Washington University St. Louis (WUSTL). The ICTS is funded by the Clinical and Translational Science Awards (CTSA) initiative, a large-scale scientific infrastructure funding program designed to enhance the quality and impact of translational research from scientific discovery to patient care [14]. The CTSA program places a strong emphasis on collaboration across disciplinary lines, a concept that researchers support [15]. For example, in our evaluation of the ICTS, investigators indicate that the transdisciplinary research improved their research conduct, productivity, and impact [16]. However, in the CTSA context, no studies have examined whether disciplinary patterns among scientists are associated with greater or less scientific collaboration.

Network analysis is the ideal analytic tool for exploring the disciplinary dynamics in team science, particularly given its use in examining transmission of scientific discovery across different groups [17]. Network analysis is also starting to be used more frequently in studies of CTSA activities. Nagarajan and colleagues [18] examined community structure variation of grant collaborations over four years at the University of Arkansas for Medical Sciences. They found non-random community structures especially in later compared to earlier years. A similar study was completed at the University of Kentucky CTSA that showed the presence of intricate research communities that were less random with more intercommunity cross talk after the CTSA was established [19]. Another CTSA study found that grant collaboration networks had small-world properties, or increased clustering compared to random graphs. They also were able to detect key scientists in the network and make predictions of new links for research collaboration [20]. The results of these studies may be visualized in powerful interactive graphs that have been developed through these studies [21]. Our own previous network study of ICTS members presented descriptive network statistics that showed increased overall collaboration in grants and publication after ICTS formation [22]. While it appeared that cross-disciplinary collaboration was more common for grant submissions compared to publications, that study did not include a statistical test of the association between disciplines and scientific collaboration.

Collectively, these studies reveal the promise and the challenges of examining interdisciplinary collaboration over time. There are many variables that may influence large scientific networks such as institutional characteristics, investigator traits, and disciplinary affiliation. All the studies discussed above are descriptive network models that have difficulty in considering such variables. Therefore, an inferential model that includes multiple variables is needed.

This paper presents an inferential network model of academic collaboration at the WUSTL ICTS. We examine the influence of investigator traits, institutional characteristics, local network structure, and discipline in grant and publication networks. This study is important to understand the factors that are associated with collaboration, with a particular focus on discipline. The paper has three aims. First, we will show the descriptive statistics of the factors and the networks. Second, we will present the inferential network model with all factors and their statistical associations to forming a collaborative tie. Third, we will use the model to show the probabilities of forming a tie under specific conditions.

## Materials and Methods

The data for this study derive from a larger evaluation of the WUSTL ICTS. The goal was network analyses of grant and publication collaborations among ICTS members. A detailed description of the data collection can be found in [22].

### Participants

The participants were ICTS members. Scientists completed an online self-registration form to become members. ICTS recruitment began in December 2007, with most initial members joining in 2008. There were 482 members by the end of 2008, and 1,272 members by the end of 2011. This study focused on groups of ICTS members who published papers or submitted grants in a given year. In other words, the 2007 (pre-ICTS) data include scientists who submitted a grant or published in 2007 and joined ICTS in 2008. The latter year networks (2010, 2011) included individuals who wrote grants or published in those respective years. This method facilitated a pre and post intervention analysis.

IRB approval was not required for secondary analysis of grant submission and publicly available publication information.

### Measures

#### Investigator demographics

Demographic variables were collected when members registered for ICTS membership. Members indicated their academic position (Professor, Associate Professor, Assistant Professor, Instructor, and other categories that were collapsed into Non-faculty for this analysis), degree (MD and/or a PhD), and institution (collapsed into WUSTL and non-WUSTL categories for the present analysis). Time of joining ICTS was the year of online registration. Members selected a single primary disciplinary specialty from a formal list of 205 hierarchical categories from the National Institutes of Health Field of Training list [23].

For statistical analysis, these categories were collapsed into four groups based on standard disciplinary groupings and similar counts in each. Each category was assigned to a single group as follows: Clinical Science (Clinical Disciplines, Predominantly Clinical Research Training categories), Allied Health (Public Health, Allied Health, Dentistry, Pediatric Disciplines, Nursing, and Veterinary Medicine categories), Basic Science (Predominantly Non-Clinical or Lab-Based Research Training, Biochemistry, Bioengineering, Biophysics, Biotechnology, Cell and Developmental Biology, Chemistry, Environmental Sciences, Genetics, Immunology, Microbiology and Infectious Diseases, Molecular Biology, Neuroscience, Nutritional Sciences, Pharmacology, Physiology, Plant Biology, Non-Clinical Radiation, and Non-Clinical Trauma categories), and Social Science (Non-Clinical Psychology, Social Sciences, and Statistics and/or Research Methods and/or Informatics categories).

#### Grant submission data

Grants data for all ICTS members by the end of 2010 were obtained from a central WUSTL administrative database. These include new submission from 2007 to 2010 including federal, state, local, and foundation grants, contracts, programs, and sub-agreements, excluding renewals, and resubmissions. Only grants submitted through WUSTL were collected because grant submission data from other institutions were not available. Given the small proportion of non-WUSTL ICTS members, omission of non-WUSTL grants was likely non-consequential.

#### Publication data

ICTS members were searched in Elsevier Scopus using the author search tool. If one author profile match appeared and if the author name was sufficiently unique, the profile was selected to display the documents for the ICTS member. Results were filtered for the desired years: 2007–2011; and publication types: articles, conference papers, reviews, and short surveys. In-press articles, books, editorials, erratum, letters, and notes were not included.

If there were multiple profile matches (including profile matches with only one document) for an ICTS member, external sources such as the ICTS membership list, LinkedIn, departmental websites, Google Scholar, and other sources were checked to locate a CV and/or affiliation information and education history. Based on information found in external resources, the appropriate author profiles from Scopus were selected and review of documents for the selected author profiles was done to manually reconcile results associated with the ICTS member.

#### Network data

The basic unit of analysis in this project was a collaboration tie. For grant collaboration ties, ICTS members were linked if they were listed as key personnel on the same contract, grant, program, or sub-agreement submission in a given year. For publications, ICTS members were linked if they were co-authors on a published article in a given year. To examine changes over time, data from two periods were examined: 1) the year prior to ICTS formation (2007) and 2) two years during ICTS activity (2010 for grants and 2011 for publications). The datasets were staggered because these were the most recent data available at the time of analysis.

### Statistical Analysis

Analyses focused on network description, visualization, and statistical modeling. Analyses for 2007 were from the investigators who registered in 2008. This was the most reasonable way to assess activities prior to ICTS formation. Analyses for 2010 and 2011 include all investigators who had signed up for ICTS membership by those years.

Network descriptive statistics were calculated for grants and publications at the two intervals. *Density* is the proportion of observed ties to the maximum possible number of ties, and indicates connectedness. The *largest component size* is the size of largest connected cluster of nodes. *Average degree* is the average number of ties per node. *Maximum degree* is the highest number of ties for a single node. *Betweenness centralization* is the variability of the betweenness centrality of network nodes, where betweenness centrality is the extent to which a node connects other pairs of nodes that are not otherwise connected. Finally, *modularity* is a chance-corrected measure of the extent to which the network’s structure is explained by cohesive subgroups, with higher scores indicating greater cohesion within groups [24]. Mathematical functions of these statistics are provided in S10 File.

Exponential random graph models (ERGMs) were conducted for 2007 grants and publications, 2010 grants, and 2011 publications. ERGMs are statistical models that predict network ties and can be interpreted in a manner similar to logistic regression while accounting for the non-independence inherent in network data [25,26]. Variables that can be used as predictors in statistical models include characteristics of network members (attributes), characteristics of the network itself (structural terms), as well as other network relationships between network members (e.g., discipline-discipline pairing) [27]. In order to determine the impact of discipline on scientific collaboration, all other demographic variables (academic position, MD, PhD, institution, and year entering ICTS) were entered into the first step of the model along with structural terms. The year entering ICTS could not be used in the 2007 models, as there was no variability in this variable among the charter members. Discipline was added in the second step as an attribute mixing term, which allowed for the examination of all possible cross- and within-disciplinary collaboration patterns among researchers (e.g., clinical with clinical, clinical with allied health). The fitted models were then used to estimate probabilities of collaboration ties given varying discipline patterns.

Descriptive network statistics and modularity were calculated using Pajek version 3.14. ERGMs and predicted probabilities were conducted using RStatnet version 2014.2.0 [28] on R version 3.1.2. To reproduce these findings, the full dataset of deidentified participants’ attributes and network edgelists are available in supporting information.

## Results

### Characteristics of Grant and Publication Networks

In the 2007 networks, 186 scientists submitted grants and 224 published papers. Almost 500 scientists submitted grants in 2010, and 833 scientists published in 2011. Table 1 shows the demographic characteristics in each of these networks. Importantly, although the networks grew over time, the ratio of scientists in each discipline remained relatively stable. The proportion of senior faculty also changed during this time; the latter years had relatively more junior faculty and less full professors in the networks. As is the case with other CTSA initiatives, biomedical clinical research was the largest group in this analysis.

**Table 1:** Investigators’ demographics by relationship and year.

	Grants				Publications			
	2007		2010		2007		2011	
	N	%	N	%	N	%	N	%
*Academic Position*								
Non-Faculty	4	2.2	24	4.9	3	1.3	79	9.5
Instructor	7	3.8	38	7.7	15	6.7	64	7.7
Assistant Professor	52	28.0	143	29.0	60	26.8	240	28.8
Associate Professor	43	23.1	118	23.9	53	23.7	184	22.1
Professor	80	43.0	163	33.1	93	41.5	253	30.4
Missing	0	0.0	7	1.4	0	0.0	13	1.6
*MD Degree*								
No	64	34.4	174	35.3	81	36.2	315	37.8
Yes	122	65.6	312	63.3	143	63.8	506	60.7
Missing	0	0.0	7	1.4	0	0.0	12	1.4
*PhD Degree*								
No	93	50.0	249	50.5	115	51.3	433	52.0
Yes	93	50.0	237	48.1	109	48.7	388	46.6
Missing	0	0.0	7	1.4	0	0.0	12	1.4
*Institution*								
Non WUSTL/BJC	12	6.5	20	4.1	17	7.6	97	11.6
WUSTL & BJC	174	93.5	466	94.5	207	92.4	724	86.9
Missing	0	0.0	7	1.4	0	0.0	12	1.4
*Year Entering ICTS*								
2008	186	100.0	193	39.1	224	100.0	234	28.1
2009			168	34.1			228	27.4
2010			132	26.8			177	21.2
2011			NA	NA			194	23.3
*Discipline*								
Clinical Science	100	53.8	261	52.9	121	54.0	451	54.1
Allied Health	29	15.6	70	14.2	37	16.5	122	14.6
Basic Science	44	23.7	131	26.6	50	22.3	213	25.6
Social Science	13	7.0	31	6.3	16	7.1	47	5.6
Total	186		493		224		833	

**Fig 1:**
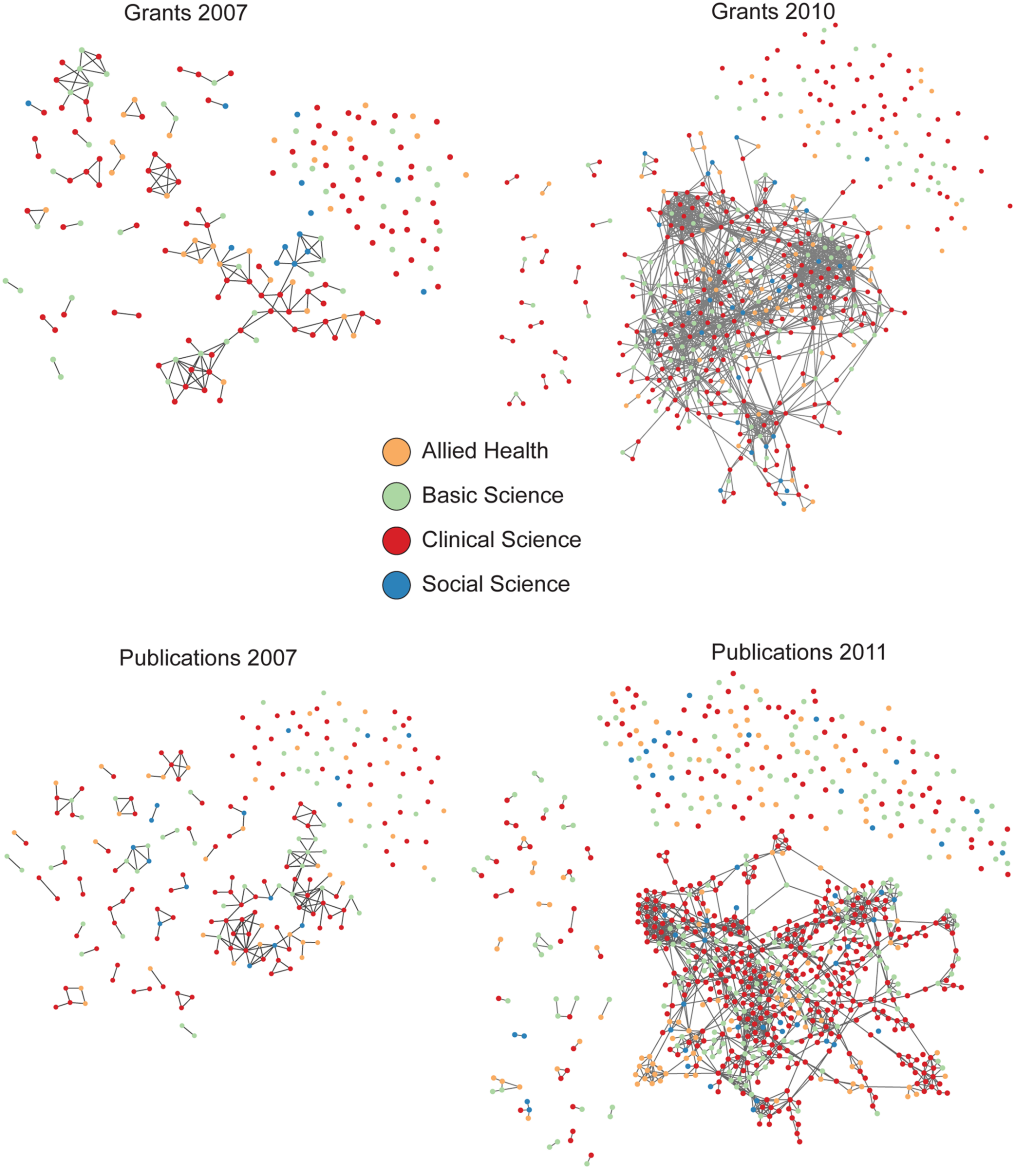
Four collaboration networks of ICTS scientists color coded by discipline.

Fig 1 shows the four networks with colors indicating the disciplinary affiliation. The growth in the number of investigators from 2007 to 2010 and 2011 is readily apparent. Note also the greater cohesion of the networks over time: both the grants and publications networks in 2007 show several disconnected components of three or more researchers, whereas there are fewer components of that size in 2010 and 2011. These characteristics are described in greater detail in Table 2.

**Table 2:** ICTS network statistics for grant and publication collaborations.

	Network Size	Density	Largest Component Size	Average Degree	Maximum Degree	Betweenness Centralization	Modularity
Grants							
2007	186	0.009	61	1.6	8	0.060	0.128
2010	493	0.011	358	5.5	39	0.081	0.061
Publications							
2007	224	0.007	67	1.6	13	0.031	0.081
2011	833	0.004	566	3.6	22	0.048	0.108

Table 2 is a set of descriptive statistics for each network. Network size, largest component size, average degree, maximum degree, and betweenness centralization all increased for both grants and publications over time. The increases for maximum degree were particularly large. Density increased for grants but decreased for publications, though lower density is not surprising given the growth of the networks. These patterns point to an increase in collaboration over time, particularly for grants. Modularity decreased for grants and increased for publications. In the case of grants, the decrease in modularity indicates an increase in the amount of cross-discipline collaboration relative to within-discipline collaboration.

### Statistical Model of Network Structures

The statistical models for publications and grants are displayed in Table 3. The goal of the statistical models was to examine the influence of discipline on collaboration above and beyond other demographic and institutional characteristics. To that end, we fit the models in two stages: 1) a structural model containing all of the demographic characteristics and local network structural terms, and 2) a discipline model that added disciplinary collaboration patterns as predictors.

**Table 3:** Results of ERGM statistical models predicting collaboration ties among ICTS members.

	Grants				Publications			
	2007^a^		2010^b^		2007^a^		2011^c^	
	Structural	+ Discipline	Structural	+ Discipline	Structural	+ Discipline	Structural	+ Discipline
Edges (constant)	-5.42 (1.36)	***-5*.*83 (1*.*45)***	***-6*.*17 (*.*28)***	***-5*.*99 (*.*28)***	***-6*.*87 (1*.*47)***	***-6*.*59 (1*.*53)***	***-4*.*35 (*.*25)***	***-3*.*93 (*.*25)***
Academic Position								
Non-faculty	Ref	Ref	Ref	Ref	Ref	Ref	Ref	Ref
Instructor	.16 (.47)	.25 (.48)	**.*59 (*.*12)***	**.*57 (*.*12)***	-.24 (.47)	-.28 (.48)	***-*.*39 (*.*07)***	***-*.*39 (*.*08)***
Assistant Professor	.23 (.40)	.30 (.42)	**.*84 (*.*11)***	**.*84 (*.*12)***	-.05 (.44)	-.12 (.44)	.08 (.05)	.08 (.06)
Associate Professor	.15 (.40)	.23 (.43)	**.*91 (*.*12)***	**.*90 (*.*11)***	.01 (.44)	-.06 (.44)	**.*21 (*.*06)***	**.*22 (*.*06)***
Professor	.39 (.40)	.46 (.42)	***1*.*10 (*.*12)***	***1*.*11 (*.*12)***	.07 (.44)	-.01 (.45)	**.*46 (*.*06)***	**.*48 (*.*06)***
MD degree	-.10 (.13)	-.05 (.13)	**.*18 (*.*04)***	**.*13 (*.*04)***	.04 (.11)	.03(.13)	**.*18 (*.*04)***	.03 (.04)
PhD degree	-.07 (.12)	-.08 (.12)	**.*14 (*.*04)***	**.*19 (*.*04)***	.02 (.11)	.02 (.11)	**.*18 (*.*04)***	**.*25 (*.*04)***
Same Institution	.05 (.20)	.05 (.20)	-.05 (.07)	***-*.*01 (*.*07)***	.35 (.22)	.34 (.23)	**.*43 (*.*06)***	**.*47 (*.*06)***
Year entering ICTS			***-*.*38 (*.*03)***	***-0*.*38 (*.*03)***			***-*.*06 (*.*01)***	***-*.*08 (*.*02)***
Structural terms								
GWD	-.38 (.53)	-.25 (.52)	***-1*.*44 (*.*31)***	***-1*.*44 (*.*32)***	.44 (.47)	.44 (.47)	***-3*.*57 (*.*21)***	***-3*.*38 (*.*20)***
GWESP	2.27 (.21)	***2*.*25 (*.*21)***	***2*.*60 (*.*08)***	***2*.*60 (*.*09)***	***2*.*34 (*.*18)***	***2*.*34 (*.*18)***	***2*.*54 (*.*06)***	***2*.*52 (*.*07)***
GWDSP	-.24 (.11)	***-*.*20 (*.*10)***	***-*.*09 (*.*01)***	***-*.*09 (*.*01)***	-.02 (.07)	-.03 (.07)	***-*.*28 (*.*02)***	***-*.*28 (*.*02)***
Discipline								
Clinical-Clinical		Ref		Ref		Ref		Ref
Allied Health-Allied Health		**.*86 (*.*21)***		**.*48 (*.*09)***		.17 (.40)		.12 (.20)
Basic Science-Basic Science		.27 (.28)		***-*.*19 (*.*09)***		.29 (.23)		***-*.*60 (*.*11)***
Social Sciences-Social Sciences		***1*.*16 (*.*27)***		-.22(.31)		.85 (.49)		**.*71 (*.*19)***
Clinical-Allied Health		.03 (.22)		***-*.*25 (*.*06)***		-.24 (.20)		***-*.*91 (*.*17)***
Clinical-Basic Science		.09 (.21)		***-*.*29 (*.*05)***		-.24 (.17)		***-*.*49 (*.*06)***
Clinical-Social Sciences		-.94 (.53)		***-*.*43 (*.*09)***		-.12 (.25)		-.13 (.09)
Allied Health-Social Sciences		-.62 (.88)		-.04 (.14)		.10 (.43)		-.98 (.64)
Basic Science-Allied Health		-1.03 (.56)		***-*.*63 (*.*10)***		***-*.*85 (*.*43)***		***-1*.*92 (*.*49)***
Basic Sciences-Social Sciences		.26 (.33)		***-*.*39 (*.*13)***		-.14 (.37)		***-*.*54 (*.*15)***
Fit								
AIC	1371	1353	12889	12514	1754	1757	14307	14153
BIC	1456	1508	13006	12717	1843	1920	14436	14379

^a^Alphas for all 2007 structural terms were set to 0.5.

^b^Alphas for the 2010 grant structural terms were set as follows: GWD = 0.1, GWESP = 0.1, GWDSP = 0.7.

^c^Alphas for the 2011 publications structural terms were set as follows: GWD = 0.5, GWESP = 0.1, GWDSP = 0.5.

Parameters significant at p < .05 denoted by bold italics.

The structural models include five covariates: academic position, MD degree, PhD degree, same institution, and year entering ICTS. Academic position was entered as a categorical term, comparing the likelihood of a connection to investigators of various positions with the non-faculty baseline. MD degree and PhD degree were also entered as main effect terms, with no MD or PhD as the baseline. The same institution term tested whether collaborations were more likely between investigators from the same institution. Year entering ICTS was entered as a quantitative term, with more recent years entered as higher values, testing whether collaborations were more likely with individuals who had joined ICTS more recently. Finally, there were the local structure terms (gwdegree, gwesp, and gwdsp). These terms add one network statistic each to the weighted degree distribution, dyadwise shared partners, and edgewise shared partners, respectively. These terms collectively account for local structural processes that have been interpreted as expansivity, transitivity, and structural equivalence [27].

The discipline model added a set of assortative and disassortive mixing terms based on scientist discipline affiliation to the first structural model. This approach adds one network statistic to the model for each possible pairing of node attribute values. This takes into account within-discipline pairings (e.g., both researchers are Allied Health), and cross-discipline pairings (e.g., one researcher is Clinical and the second is Allied Health).

Academic position was more important in the latter years (2010 grants, 2011 pubs), with collaborations being most likely with professors. Having an MD or PhD also generally increased the likelihood of collaborations. The results for same institution were mixed, with investigators being more likely to publish with those from the same institution than from a different institution, but somewhat less likely to submit a grant with someone from the same institution in 2010 when discipline is added to the model. Researchers who joined ICTS earlier were more likely to collaborate than those who joined later for both grants and publications

Overall, the addition of the scientists’ discipline improved the fit of the models noted in the last two lines of the table, except for 2007 publications. The influence of discipline is more important in the latter years. For grants, only two of the mixing patterns were significant in 2007 with seven significant in 2010. For publications, one of the patterns was significant in 2007 and six were significant in 2011. Generally speaking, most discipline pairings were less likely than the clinical-clinical pairing with the exception of allied health-allied health, which was greater (significantly so in the grants models).

### Estimation of Collaboration Ties

The individual parameter estimates for the various mixing terms are difficult to interpret in isolation. Therefore, to better understand the pattern of collaborations, the fitted models were used to forecast the probabilities of collaboration ties across disciplines.

**Fig 2:**
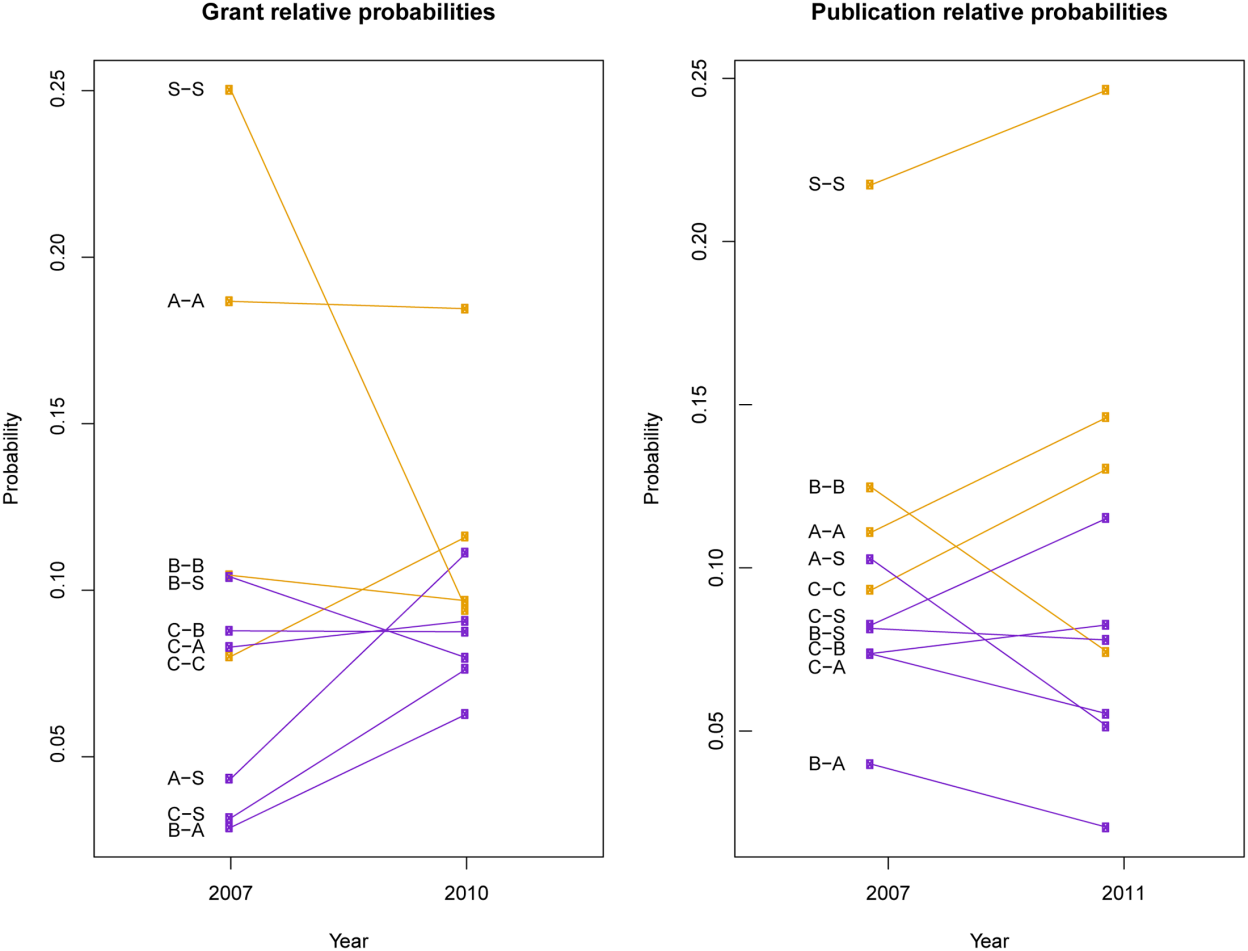
Ladder plots showing predicted changes in cross- and within-discipline collaboration for grants and publications. Cross-discipline changes are shown in purple, and within-discipline changes are shown in orange. Disciplines are coded as follows: C-Clinical, B-Basic sciences, A-Allied health, S-Social sciences.

To focus on the influence of disciplinary combinations on collaboration, all variables were held constant as the following: two individuals who were both at the rank of professor, one with a Ph.D. and the other with a M.D., both from the same institution, and with average local network structure (e.g., degree, shared partners). Predicted probabilities were estimated across all possible disciplinary combinations of collaboration using the method described in [25]. Raw and relative probabilities were calculated for each discipline pairing, and relative probabilities are shown in Fig 2. Because there was an overall increase in the total amount of collaboration in the later years, relative probabilities were used to compare patterns over time. A cross-disciplinary ratio was then calculated, which was the sum of all cross-disciplinary combinations divided by the total. This ratio measures the extent to which the total collaboration pattern was cross-disciplinary.

For grants, the cross-disciplinary ratio moved from .38 in 2007 to .51 in 2010, an increase of 34%. For publications, the ratio decreased by 11% from .45 in 2007 to .40 in 2011. Fig 2 shows the specific changes in relative probability of collaboration, separated by cross-discipline (purple) and within-discipline (orange) relationships. For grants, the likelihood of cross-discipline collaboration increased for five out of six, and within-discipline collaboration decreased in three out of four. This pattern was not seen in the publication forecasts. The likelihood for within-discipline collaboration increased for three out four, and cross-discipline collaboration decreased for five out of six. Overall, these patterns suggest that grant collaboration was increasingly a cross-discipline activity, while publications remained primarily a within-discipline activity.

## Discussion

Disciplinary affiliation independently predicted collaboration in grant and publication networks, particularly in the later years. The statistical model incorporated demographic, institutional, and network structure covariates, and it was robust across four observed networks. Overall collaboration increased in post-ICTS networks, with cross-discipline ties occurring more often than within-discipline ties in grants, but not publications.

In our review of the science of team science studies, this paper is the first to use a multivariate network model that shows the importance of discipline in scientific networks. Bibliometric studies have noted the tendency for collaboration across certain disciplines and within institutions [9, 10]. Other network studies on CTSA research communities have described collaboration dynamics over time [19, 29]. However, this report illustrates that disciplinary patterns are robust covariates in a statistical model accounting for some, but not all, factors associated with collaboration. Importantly, this research does not confirm a causal relationship between discipline and collaboration. There are multiple environmental factors during this period that could not be measured such as funding climate, faculty appointment changes, and infrastructure development at WUSTL.

From a wider policy perspective, such models may be used to evaluate discipline linkage patterns, and promote cross-disciplinary teams. For example, Vacca and colleagues [30] show the feasibility of altering the collaboration network in a CTSA-funded institution by introducing previously unconnected investigators. Such a network intervention could enhance desirable structural properties for the individual scientist and the community as a whole. Their results suggest the promise of such models to inform and catalyze scientific innovation using a network approach.

A second policy-related implication is the pattern of cross-discipline ties in grants and within-discipline ties in publication. As Gewin [31] observed, this may reflect a growing disconnect between funding and publication cultures. The former encourages exuberant proposals with team members from differing specialties. The latter has more discipline-focused criteria evaluated by specialized editorial boards. Certain academic departments also employ implicit or explicit promotion criteria that reward within-discipline contributions. Such criteria may particularly impact the collaboration patterns of junior faculty who increased in numbers between the early and late years in the ICTS networks. Another explanation is that paper co-authorship is a lagging indicator of collaboration. If this were true, then cross-discipline co-authorship would be expected to increase in subsequent years. We will test these hypotheses in upcoming analyses.

There were limitations to this study. We did not have full data on the pre-ICTS research community. Therefore, the 2007 pre-ICTS data were extrapolated from the first cohort who entered ICTS, which may be a source of bias. Second, collaborating investigators who did not enroll in the WUSTL ICTS until later years may artificially increase the collaboration ties when they were included in the network with ‘new’ ties to existing members. Third, environmental factors such as funding climate and infrastructure development that may influence collaboration could not be included in the models. Finally, this study does not examine collaboration change related to discipline over more than two slices in time, nor the influence of grant network patterns on publication networks. We are currently studying both of these areas using stochastic actor-based models.

This study showed the importance of disciplinary affiliation as an independent factor in collaboration patterns over multiple years. It re-affirms the network approach as central to the study of team science, and introduces the use of exponential random graph models as a next step after descriptive network studies in this domain. These are still early moments in understanding professional collaborations, particularly in terms of the factors influencing its development and evolution in a non-reductionist manner. Nonetheless, these results are important to inform better evaluation and interventions of academic collaboration.

## Supporting Information

S1 File Attribute Codebook. Codebook for the raw data files.
(PDF)

S2 File Grants 2007 Attributes. De-identified participants’ attributes for 2007 grants network.
(CSV)

S3 File Grants 2010 Attributes. De-identified participants’ attributes for 2010 grants network.
(CSV)

S4 File Publications 2007 Attributes. De-identified participants’ attributes for 2007 publications network.
(CSV)

S5 File Publications 2011 Attributes. De-identified participants’ attributes for 2011 publications network.
(CSV)

S6 File Grants 2007 network. Pajek file with network vertices and edgelist for 2007 grants network.
(NET)

S7 File Grants 2010 network. Pajek file with network vertices and edgelist for 2010 grants network.
(NET)

S8 File Publications 2007 network. Pajek file with network vertices and edgelist for 2007 publications network.
(NET)

S9 File Publications 2011 network. Pajek file with network vertices and edgelist for 2011 publications network.
(NET)

S10 File Network statistics. Mathematical functions of network statistics used in the paper.
(PDF)
