# Supplementary material for: Academic Cross-Pollination: The Role of Disciplinary Affiliation in Research Collaboration
Source: PLoS One. 2016 Jan 13;11(1):e0145916. doi: 10.1371/journal.pone.0145916 (PMC4711942; doi:10.1371/journal.pone.0145916)
Supplement: S1 File — Codebook for the raw data files. (PDF) [file pone.0145916.s001.pdf]

Node Attribute Codebook

Academic Position

Value	Label
1	Non-Faculty
2	Instructor
3	Assistant Professor
4	Associate Professor
5	Professor
9999	Missing

MD, PhD

Value	Label
0	No
1	Yes
9999	Missing

Institution

Value	Label
0	Non WUSTL/BJC
1	WUSTL & BJC
9999	Missing

Discipline

Value	Label
1	Clinical Science
2	Basic Science
3	Allied Health
4	Social Science

Note: Attributes are stored in .csv files. The corresponding networks are stored in Pajek .net files.
