# Supplementary material for: Academic Cross-Pollination: The Role of Disciplinary Affiliation in Research Collaboration
Source: PLoS One. 2016 Jan 13;11(1):e0145916. doi: 10.1371/journal.pone.0145916 (PMC4711942; doi:10.1371/journal.pone.0145916)
Supplement: S10 File — Mathematical functions of network statistics used in the paper. (PDF) [file pone.0145916.s010.pdf]

Density [1]:

$$\Delta = \sum \frac{C_D(n_i)}{g(g-1)}$$

Average Degree [1]:

$$\overline{C}_D = \sum \frac{C_D(n_i)}{g}$$

Betweenness Centrality [1]:

$$C'_B(n_i) = \frac{\sum_{j<k} \frac{g_{jk}(n_i)}{g_{jk}}}{\frac{(g-1)(g-2)}{2}}$$

Betweenness Centralization [1]:

$$C_B = \frac{\sum_{i=1}^{g} [C'_B(n^*) - C'_B(n_i)]}{g-1}$$

Modularity [2]:

$$Q = \sum_i (e_{ii} - a_i^2)$$

Where:

$C_D(n_i)$ = number of links an actor has in the network

g = number of actors in the network

g_{jk} = number of shortest paths connecting two actors

$g_{jk}(n_i)$ = number of shortest paths connecting two actors that include actor *i*

$C'_B(n^*)$ = largest betweenness centrality in the network

e_{ii} = fraction of links connecting actors in the same discipline

a^i = fraction of links connecting to actors in discipline *i*

[1] Wasserman S, Faust K. Social network analysis: Methods and appilcations. Cambridge: Cambridge Univsity Press; 1994..

[2] Newman MEJ, Girvan M. Finding and evaluating community structure in networks. Phys Rev E Stat Nonlin Soft Matter Phys. 2004. doi: 10.1103/PhysRevE.69.026113
